# School eHealth Education Program Pakistan (eSHEPP) to improve NCDs awareness in adolescents from urban Pakistan: a mixed method design protocol

**DOI:** 10.1186/s41043-025-01097-6

**Published:** 2025-10-14

**Authors:** Muhammad Shahid Khan, Aysha Almas, Zainab Samad, Kanecia Obie Zimmerman, Tazeen Saeed Ali

**Affiliations:** 1 Department of Medicine, Aga Khan University, Karachi, 74800 Pakistan; 2 Department of Pediatrics, Duke University Medical Center, Duke University, Durham, 27710 USA; 3 Duke Clinical Research Institute, Duke University School of Medicine, Duke University, Durham, 27710 USA; 4 School of Nursing and Midwifery, Aga Khan University, Karachi, 74800 Pakistan; 5 Department of Community Health Sciences, Aga Khan University, Karachi, 74800 Pakistan

**Keywords:** Non-communicable diseases, Adolescents, EHealth, School-based intervention, Pilot cluster randomized controlled trial, Knowledge, Attitudes, Practices

## Abstract

**Background:**

Non-communicable diseases (NCDs) pose a significant health threat, particularly among adolescents in low- and middle-income countries (LMICs) such as Pakistan. Unhealthy habits, including poor diet, physical inactivity, and substance use, often begin in childhood. These behaviors not only increase the risk of NCDs later in life but can also cause serious health problems during adolescence or early adulthood. This protocol aims to assess the feasibility and potential efficacy of the School eHealth Education Program Pakistan (eSHEPP) in improving the knowledge, attitudes, and practices (KAP) of school students in Karachi regarding NCDs and their risk factors.

**Methods and analysis:**

A sequential mixed-methods design will be used, combining qualitative and quantitative approaches. In Phase 1, focus group discussions (FGDs) and interviews with stakeholders will identify barriers and facilitators to implementation, as well as gather perspectives on the eHealth application’s design and content. In Phase 2, a pilot cluster randomized controlled trial (cRCT) will assess the program’s feasibility and potential efficacy in improving participants’ KAP regarding NCDs. In Phase 3, FGDs with intervention participants will explore the program’s usability, acceptance, and task–technology fit.

**Participants:**

The study will engage a diverse group of stakeholders across three phases. Phase 1 will involve four FGDs, two with students and two with teachers (eight participants per FGD), and ten key informant interviews (five with parents and five with school administrators). In Phase 2, 272 secondary and higher secondary school students aged 13–18 years will be recruited from selected schools in Karachi. Phase 3 will include two FGDs with students (eight participants per FGD). Written assents and consents will be obtained from all participants.

**Intervention:**

The eSHEPP will deliver digital health education via an interactive eHealth application to raise awareness about NCDs and their risk factors among adolescents. Students in the intervention group will attend health-promoting sessions in the classroom for over two months, while the control group will receive no intervention and continue with the routine teaching schedule.

**Outcomes:**

Phase 1 will identify barriers and facilitators to implementing the eSHEPP in secondary and higher secondary schools and explore stakeholders’ perceptions of its design and content. Phase 2 will assess feasibility, defined as achieving recruitment, retention, and treatment fidelity rates above 70%, and potential efficacy, indicated by a ≥ 25% improvement in KAP after two months. Phase 3 will evaluate the program’s acceptability, perceived usefulness, and task–technology fit to determine how well it meets students’ needs and expectations in the school setting.

**Ethics and dissemination:**

Ethical approval has been obtained from the Aga Khan University Ethical Review Committee (Ref: 2023-9277-27367) and the National Bioethics Committee Pakistan (Ref: 4–87/NBCR-1089/23/1896). Findings will be disseminated through peer-reviewed journals and national and international conferences.

**Trial registration number:**

NCT06674798.

**Supplementary Information:**

The online version contains supplementary material available at 10.1186/s41043-025-01097-6.

## Strengths and limitations of this study

The sequential mixed-methods design will enhance understanding and credibility of the eSHEPP through data triangulation.

The pilot cRCT will rigorously evaluate the program while controlling for confounders, strengthening internal validity.

Diverse stakeholder perspectives will enrich the data by highlighting barriers, facilitators, and potential efficacy.

Findings may not be generalizable to educational settings or cultural contexts beyond Karachi, Pakistan.

The small number of clusters (*N* = 4) limits statistical power; therefore, analyses are exploratory, and results should be interpreted with caution.

**Supplementary Information:**

The online version contains supplementary material available at 10.1186/s41043-025-01097-6.

## Introduction

Non-communicable diseases (NCDs) and their associated risk factors represent a major global public health challenge [1], particularly in low- and middle-income countries (LMICs), where they account for a disproportionately high burden of mortality and morbidity [2]. Children and adolescents are equally susceptible to NCDs [3], and a significant proportion of premature adult deaths stem from health behaviors that begin during these stages [4, 5]. The World Health Organization (WHO) has warned of the growing public health concern of NCDs among adolescents [6]. Adolescence, a critical developmental stage marked by physical, psychological, and social changes, offers an opportunity to establish healthy behaviors that prevent adult health problems [7–9]. Risk behaviors such as physical inactivity, substance use, and poor nutrition, along with factors like overweight and obesity, contribute to the development of diseases and adverse health outcomes later in life [5].

In Pakistan, as in other LMICs, adolescents face multiple NCD-related risk factors, including low fruit and vegetable intake [10], obesity [11], sedentary lifestyles [12, 13], tobacco use [14, 15], and growing concerns about mental health, including anxiety and depression [16, 17]. Pakistan, the world’s fifth most populous country (>220 million), faces rising burdens of cardiovascular diseases, mental disorders, and diabetes [18, 19]. Nearly 45% of the population is under 18 [20], underscoring the need to invest in adolescent health [21]. Despite rising literacy and school enrollment, school-based health education remains underdeveloped, though expanding access provides an opportunity for embedding health promotion [22–24].

School-based health education is widely recognized as effective for young people, and the WHO’s framework offers guidance for tailoring interventions to adolescents [25]. Studies emphasize equipping students with decision-making skills through evidence-based strategies to improve adolescent health [26]. Despite policy recognition, Pakistan lacks a coordinated youth health program [27]. In resource-constrained settings, schools remain a key platform for health promotion, while rising mobile and internet access [28] creates opportunities to engage adolescents digitally.

Evidence from LMICs demonstrates the promise of school-based programs in reducing NCD risks. In Pakistan, the 2023 School Health Education Program (SHEPP) piloted a 10-month intervention combining health education, physical activity, teacher training, and parental engagement, achieving high recruitment and improvements in physical activity, diet, and cardiometabolic outcomes [29]. Similarly, a quasi-experimental study in Rawalpindi (2022) based on the socio-ecological model reported significant gains in physical activity [30]. These efforts parallel international initiatives such as Malaysia’s MyHeart Beat project, which used environmental interventions to improve school diets [31]. A Recent editorial also emphasized schools’ critical role in shaping lifelong health behaviors and the need for innovative approaches in LMICs [32].

Digital platforms offer innovative, accessible, and cost-effective approaches to promoting healthy diets, physical activity, and NCD prevention [33–36], particularly where traditional health services are limited [37]. With widespread mobile and internet access, tools such as apps, messaging, and peer networks have shown promise in engaging adolescents [23, 24, 35, 38–41]. A systematic review of 12 RCTs found mobile app users achieved better dietary, physical activity, and lifestyle outcomes than non-users, highlighting the effectiveness of digital interventions [42]. Evidence from LMICs, including Pakistan, further underscores their potential to address adolescent health gaps [37, 38, 40, 41, 43–46].

Feasibility assessment is essential to inform scalability of digital interventions, particularly in LMICs where delivery challenges are common [29, 47, 48]. Pilot studies often use benchmarks of ~ 70% for recruitment, retention, and fidelity, while school-based or low-resource digital interventions typically yield 10–30% improvements in knowledge, attitudes, and practices (KAP) [49–51]. Mixed-methods approaches enrich feasibility assessments by adding contextual insights and cultural relevance [52–54], and frameworks such as the Technology Acceptance Model (TAM) and Task-Technology Fit (TTF) support evaluation of usability, uptake, and sustainability [55–57].

Despite global advances in digital health, Pakistan lacks rigorously evaluated, school-based digital interventions designed specifically for adolescents. Research on effective, evidence-based strategies in this area remains limited, leading to unmet health education needs [58]. Schools offer an ideal platform to address these gaps by promoting health, raising awareness, and encouraging long-term behavior change [59, 60]. Therefore, in response to the issues discussed earlier, this study aims to assess the feasibility and potential efficacy of the eSHEPP intervention in improving adolescents’ KAP related to NCDs and their associated risk factors.

## Aims

The proposed research study aims to:

1. Identify barriers and facilitators in developing and implementing eSHEPP to enhance the KAP on NCD prevention in secondary and higher secondary school students in Karachi, Pakistan.
2. Assess the feasibility and potential efficacy of the eSHEPP in improving the KAP regarding NCDs and their associated risk factors in secondary and higher secondary school students in Karachi, Pakistan.
3. Explore the perceptions of students regarding the usefulness, acceptability, and task-technology fit of the eSHEPP in improving the KAP of Pakistani secondary and higher secondary school students regarding NCDs and their associated risk factors.

## Methods

### Study design

**Fig. 1:**
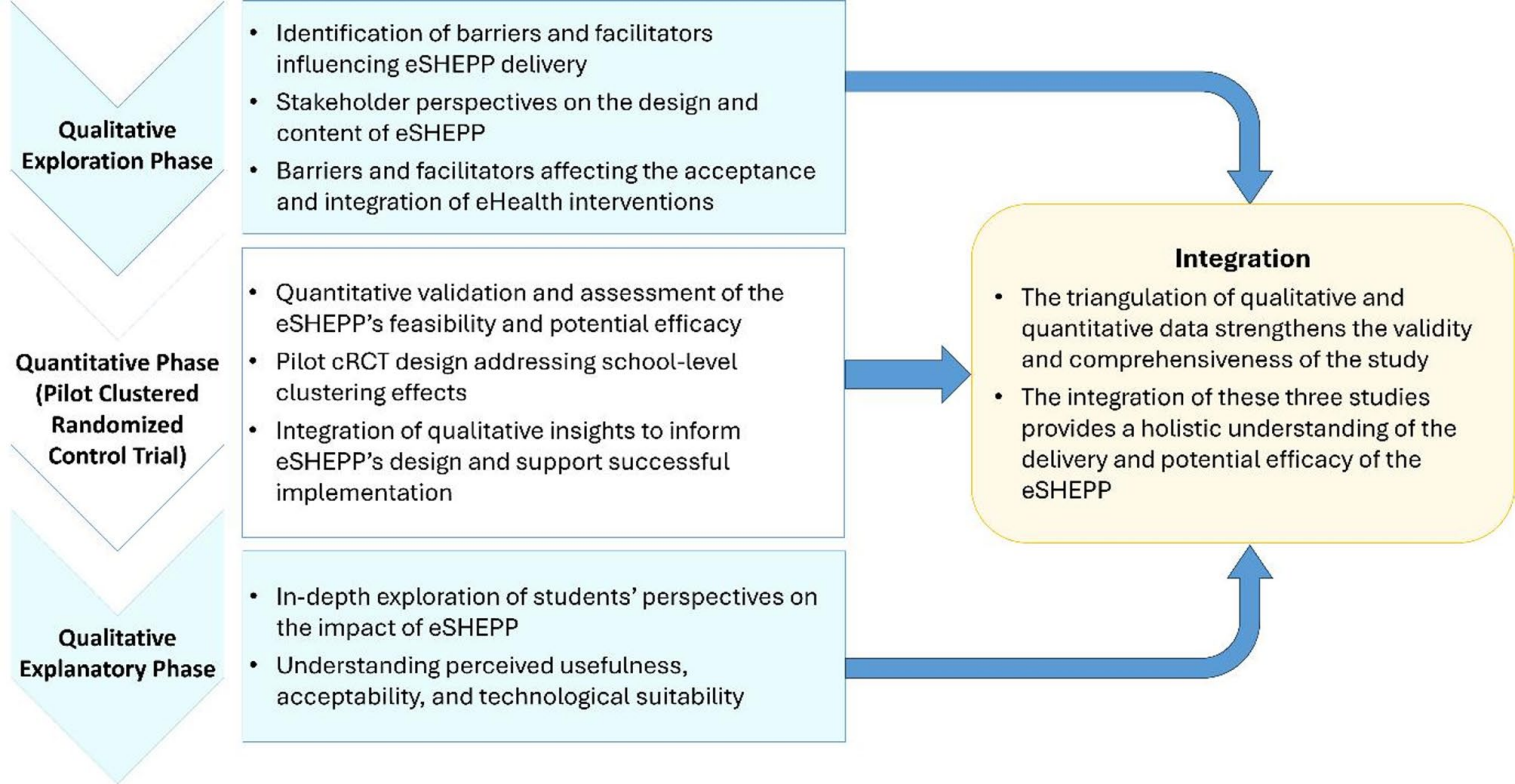
Study design of eSHEPP mixed-methods protocol

A sequential mixed methods design has been selected for this study as it combines qualitative and quantitative approaches to provide a comprehensive understanding of the eSHEPP digital application. The design begins with qualitative exploration, followed by pilot cluster randomized controlled trial (cRCT) (quantitative validation), and concludes with a qualitative explanation phase. This progression enhances the study’s rigor, allowing each phase to inform and enrich the next, while triangulating findings to strengthen the validity and robustness of the outcomes (Fig. 1).

### Study setting and study participants

The study will be conducted in Secondary and Higher Secondary Schools in the District East and District Central, Karachi, Pakistan. Secondary schools serve students in grades 9 and 10 (ages 13–15), while higher secondary schools serve grades 11 and 12 (ages 15–18) [61]. These schools provide the level of education after primary education and before higher education at the college or university level, making them ideal settings for assessing the eSHEPP intervention among adolescents. Details on the selection and expected number of participants for each phase are provided in the respective methods sections.

### Conceptual framework for eSHEPP digital application

**Fig. 2:**
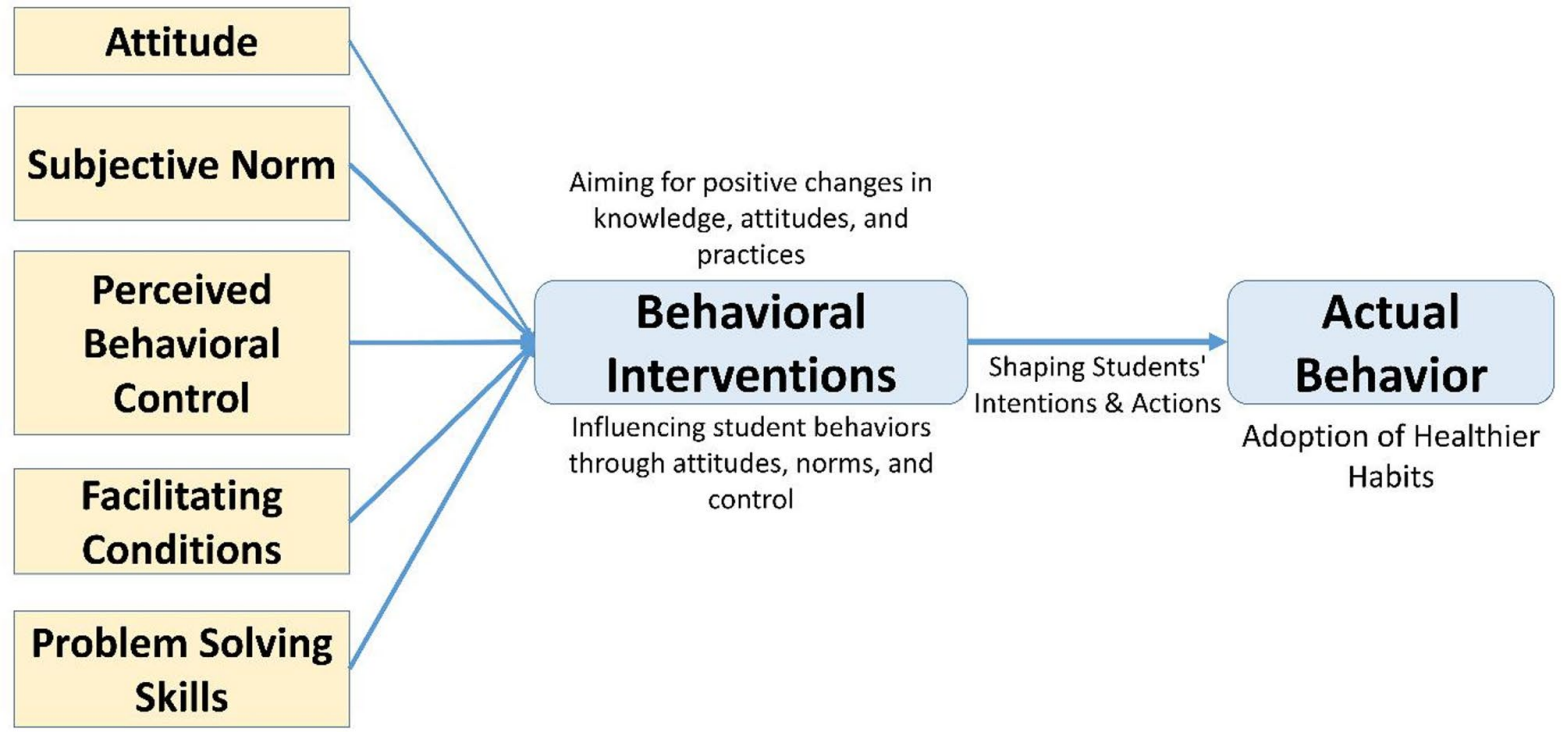
Conceptual framework for the eHealth app development

The eSHEPP digital application is guided by the Theory of Planned Behavior (TPB) [62], which examines the relationships among attitudes, subjective norms, perceived behavioral control, and intentions, all of which influence behavior. In the context of the eHealth program, students’ attitudes towards NCD prevention, societal expectations, and their self-perceived control will shape their intentions and actions in adopting healthier habits (Fig. 2).

### Development of the eSHEPP digital application - user centered approach

**Fig. 3:**
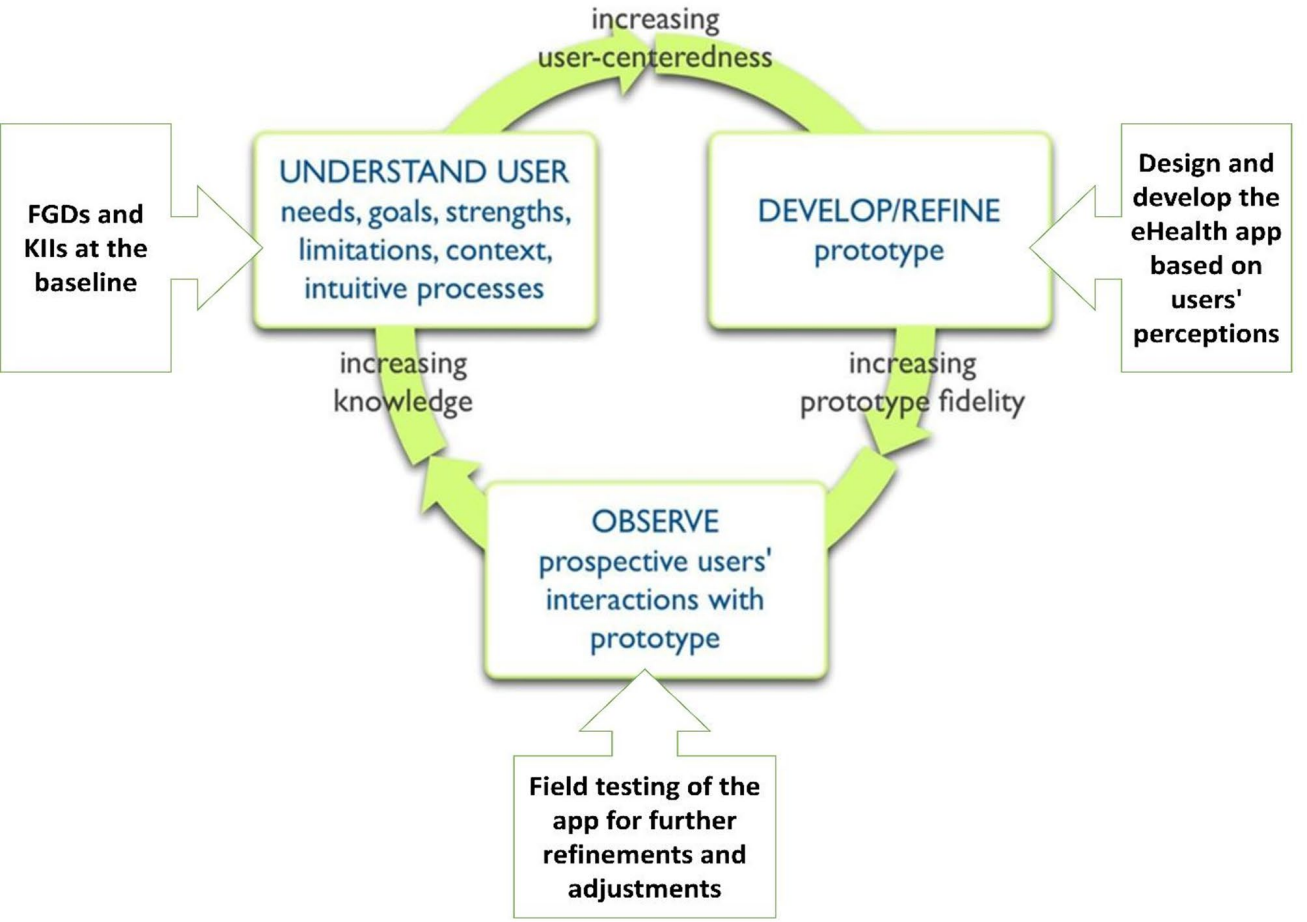
School based eHealth app - user centered approach

To ensure eSHEPP meets the target population’s needs, it will be developed using a user-centered approach (Fig. 3). This method focuses on prioritizing the needs, preferences, and experiences of users (students, parents, teachers, and other stakeholders) to create an intuitive, user-friendly application that meets their specific requirements [63].

The user-centered approach in this study consists of several key components designed to ensure the intervention is relevant, effective, and responsive to the needs of adolescents. First, understanding user needs is prioritized through initial qualitative inquiries, including interviews and focus groups, to gather insights into users’ goals and challenges (as outlined in Objective 1). This is followed by an iterative design process in which continuous feedback from stakeholders informs modifications and improvements. Prior to full implementation, the program will undergo field testing to identify any usability issues and assess its effectiveness in real-world school settings. Finally, feedback from this testing phase will guide the final adjustments to ensure the program aligns with user expectations and supports the broader goal of NCD prevention among adolescents.

By prioritizing user engagement and feedback throughout the development process, the eSHEPP aims to enhance its relevance and potential effectiveness in improving KAP on NCD prevention. This process is expected to support the successful promotion of healthy behaviors among students. Details of the full development process for eSHEPP will be reported separately.

### Proposed content of school eHealth education program Pakistan (eSHEPP)

The eSHEPP is a digital school-based health intervention that promotes healthy behaviors among secondary and higher secondary school students (ages 13–18) in Pakistan. The program emphasizes the importance of healthy diet, physical activity, and NCD prevention. Previous studies have demonstrated the effectiveness of similar school-based interventions in encouraging healthy lifestyle changes, such as improved dietary habits and increased physical activity, thereby reducing the risk of non-communicable diseases [29, 60, 64]. Previous research on the School Health Education Program Pakistan had been found feasible in improving physical activity among schoolchildren and has informed the development of the proposed eSHEPP content [29]. The eSHEPP will be implemented in partnership with the Directorate of School Education and delivered across selected public secondary and higher secondary schools in Karachi.

The program will be delivered exclusively through a custom-built digital health application, developed specifically for this initiative and accessible via standard web browsers. This dedicated platform will host all educational content and assessments, ensuring a secure and consistent learning experience. The eSHEPP will consist of three main segments. The first segment, the pre-assessment, will assess students’ baseline KAP of Non-Communicable Disease (NCD) prevention. The second segment, health education, will deliver six video-based sessions in Urdu, tailored for Pakistani secondary and higher secondary students (ages 13–18). These sessions will cover key topics, including understanding NCDs, adopting healthy eating habits (Eat Smart), engaging in regular physical activity (Keep Moving), avoiding smoking and drugs (Run Away from Smoking and Drugs), and promoting overall wellness (Stay Well). Finally, the post-assessment segment will require students to complete a post-assessment identical to the pre-assessment. Scores from the pre- and post-assessments will be displayed to highlight progress following the intervention.

### Phase I: qualitative exploration - barriers and facilitators in developing and implementing eSHEPP

#### Study design and participants

This study will utilize an exploratory qualitative study design to investigate the barriers and facilitators in implementing the eSHEPP and to assess stakeholders’ perceptions regarding the design and content of the program. For the identification of barriers and facilitators in developing and testing the eSHEPP, we will use a framework adapted from the Technology Acceptance Model (TAM) and the Task Technology Fit (TTF) [55] for the qualitative exploration and explanation phases.

The study will gather insights and perspectives from the diverse stakeholders to comprehensively understand the research topic. The study will target students in grades 9–12, teachers with at least 6 months of experience in secondary and higher secondary schools in Karachi, parents or guardians of these students, and school administrators (principals and vice principals), District Education Officers, and Provincial Department of Education decision-makers.

#### Sampling technique

Participants for Focus Group Discussions (FGDs) and Key Informant Interviews (KIIs) will be approached through the secondary and higher secondary school principals and administrators. The study will include 2 FGDs each with students and teachers, with 8 participants per FGD. Additionally, 5 KIIs will be conducted with parents and 5 with school administrators. Data collection will continue until saturation is reached.

#### Data collection and plan of data analysis

**Fig. 4:**
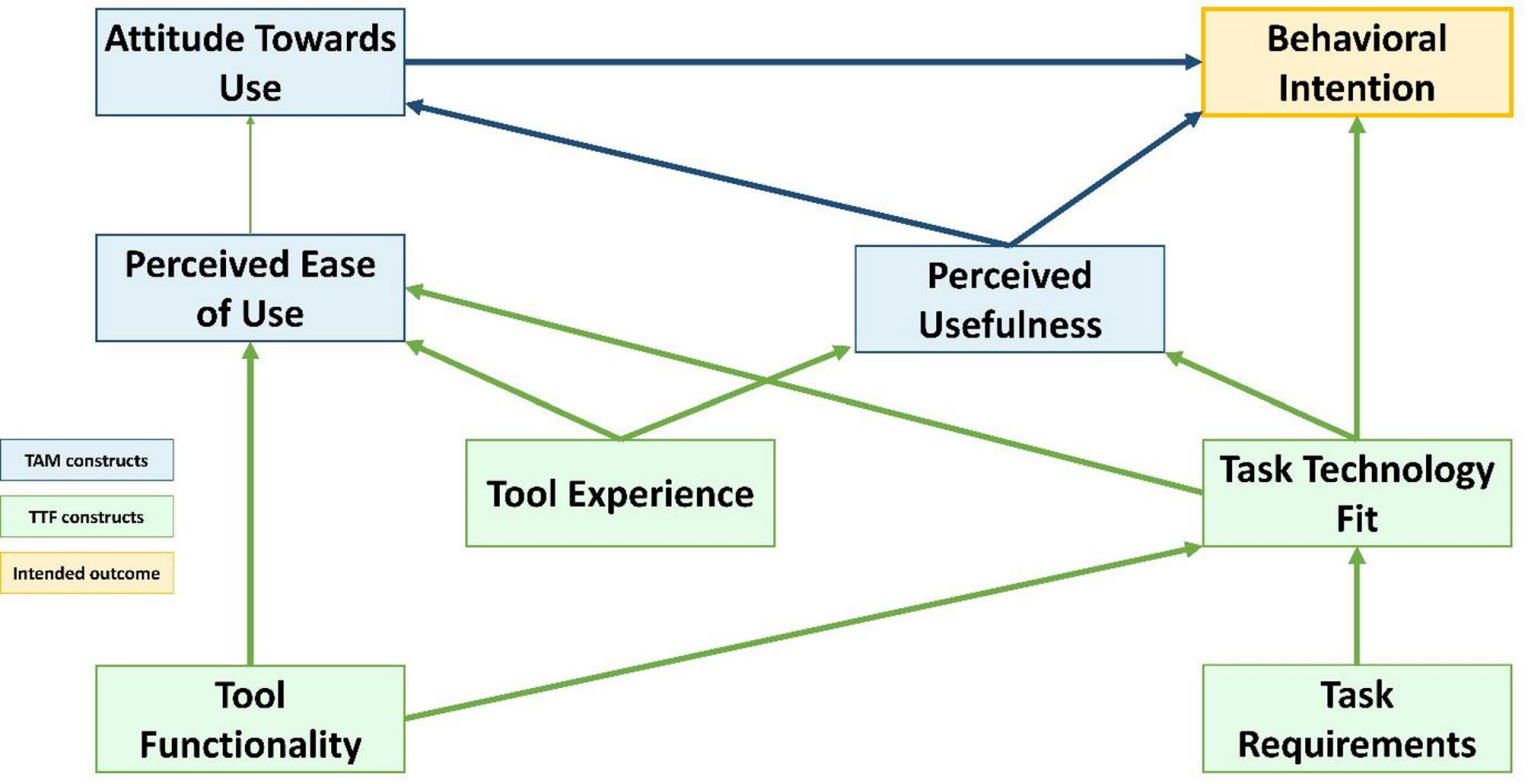
Integrated model of TAM and TTF

Semi-structured interview guides will be developed using the integrated TAM and TTF framework (Fig. 4), with specific probes informed by the model. Guides will be prepared in English and translated into Urdu to accommodate participant preferences. To ensure validity and effectiveness, a pre-test will be conducted with one FGD and one KII, and necessary adjustments will be made based on participant feedback. FGDs and KIIs will explore stakeholders’ views on barriers and facilitators for eSHEPP implementation, along with program content and design. Sessions will be facilitated by a trained qualitative researcher with experience in school-based studies. Informal conversations will be held before each session to establish rapport and clarify study objectives.

Data will be analyzed according to COREQ guidelines, combining deductive analysis (guided by TAM and TTF themes) with inductive analysis for emerging themes. Audio recordings will be transcribed verbatim, translated into English, and analyzed using thematic analysis in QSR NVivo (version 10). Verbatim quotes will be included to illustrate key findings.

### Phase II: quantitative assessment - pilot cRCT of eSHEPP vs. usual care group

#### Study design and participants

The study will be a pilot cRCT with four schools randomly assigned to the eSHEPP intervention and four schools to the control group. The target participants will be secondary and higher secondary school students from District East and District Central in Karachi, Pakistan. The sampling frame will comprise all eligible public secondary and higher secondary schools in these two districts.

#### Randomization and blinding

**Fig. 5:**
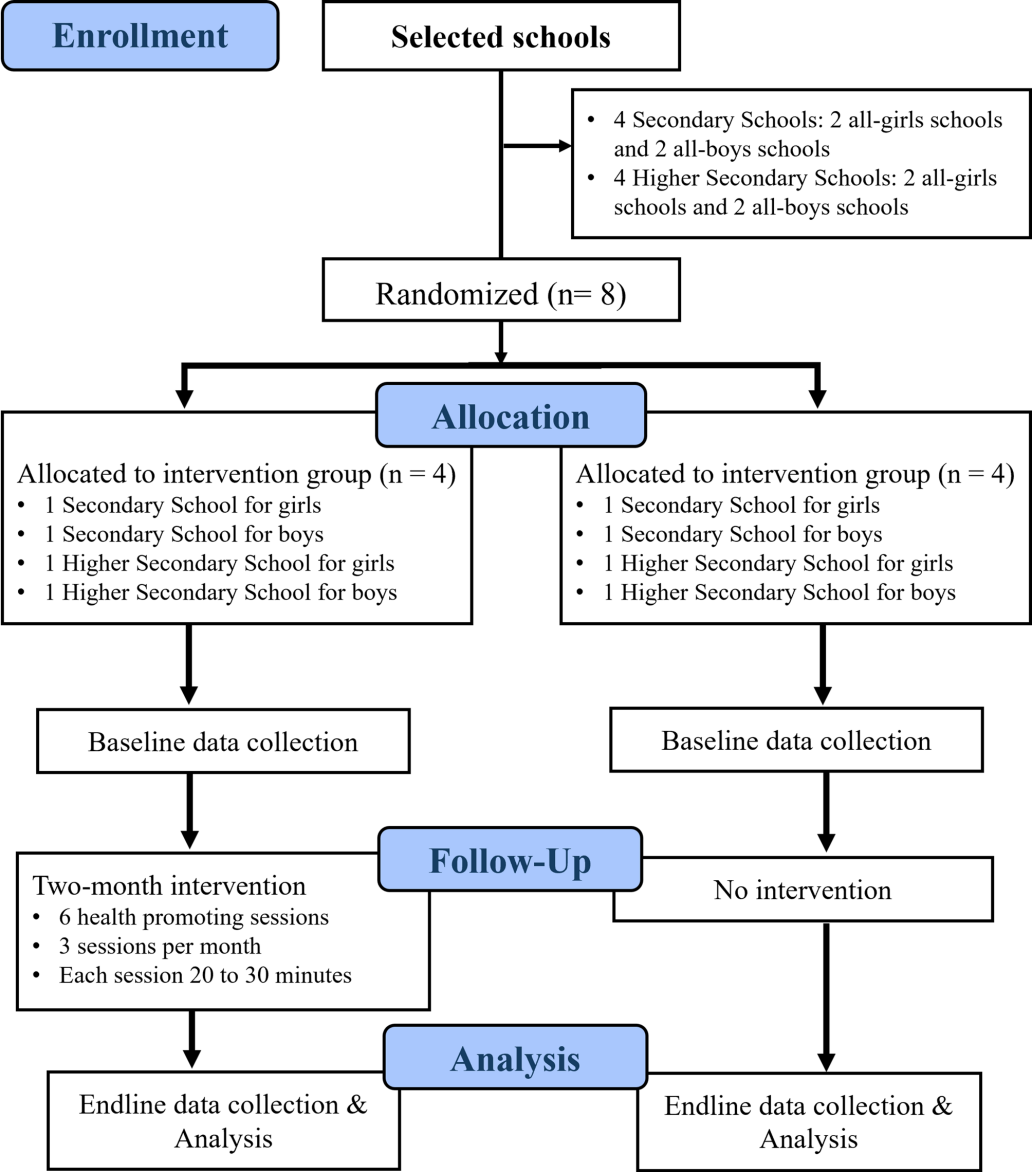
CONSORT flow diagram of the pilot cRCT

The randomization sequence will be generated using a computer-based random number generator, and allocation will be concealed until the intervention begins to prevent bias. An independent researcher will oversee the process for transparency and error minimization, as illustrated in the CONSORT flow diagram of the pilot cRCT (Fig. 5). Blinding of participants, facilitators, and researchers is not feasible due to the interactive nature of eSHEPP, as both students and facilitators will be aware of their group assignments.

#### eSHEPP intervention

Before randomly assigning schools to intervention and control groups, baseline data on students’ awareness will be collected. Allocation will be revealed after recruitment and baseline assessments to prevent recruitment bias. The eSHEPP intervention will be a digitally delivered program comprising six multimedia-based health education sessions (20–30 min each) over two months. Sessions will be delivered through an interactive eHealth platform, incorporating videos, animations, quizzes, and infographics designed to engage adolescents and promote learning. Facilitators will oversee digital delivery, ensure content accuracy, and monitor student engagement. The platform’s built-in analytics will enable real-time monitoring of student activity, participation, and completion rates. The control group will receive no intervention during the study but will undergo an endline assessment after two months. They will then receive one condensed session on NCDs and their risk factors.

#### Sample size

The sample size for this pilot cluster randomized controlled trial (cRCT) was determined using the following assumptions: four clusters (two intervention and two control schools) with an average of 300 students per cluster, an intra-cluster correlation coefficient (ICC) of 0.025 [65], statistical power of 80%, a two-sided significance level of 0.05, and an expected effect size of 25% improvement in KAP related to NCDs. Based on these parameters, the minimum required sample size was estimated to be 247 participants. To account for a potential 10% attrition rate, the final sample size was adjusted to 272 participants, with 136 students allocated to each group. This sample size is considered adequate to detect the anticipated effect size while accounting for clustering effects. The calculation was conducted using JupyterHub software [66].

#### Data collection tool and procedure

The study will adapt a validated self-administered questionnaire originally developed to assess the KAP of adolescents in Bangladesh regarding NCDs and their behavioral risk factors [67]. The development of this questionnaire involved a comprehensive review of existing survey instruments used in the field, including the Global School Health surveys, STEPs Survey of NCD risk factors, and a Mongolian survey questionnaire. The GSHS has shown moderate test-retest reliability (Cohen’s kappa = 0.47) in some contexts, notably for tobacco and alcohol items [68]. The STEPS tool has demonstrated good reliability (ICC >0.70) across LMICs [69, 70]. Although limited psychometric data are published for the Mongolian version, it is based on the STEPS framework and has shown acceptable internal consistency (Cronbach’s alpha = 0.70) in related settings [71, 72]. These instruments formed the basis of the original Bangladeshi questionnaire being adapted in this study, and were selected for their global relevance, applicability to adolescent health-risk behavior research, and proven adaptability to diverse and low-resource environments, supporting reliable data collection and methodological consistency.

As part of the adaptation process, five subject experts will review the questionnaire and recommend revisions to ensure it is culturally and contextually tailored to the target population. The questionnaire will then be pre-tested on a similar population to gather feedback and refine it for effectiveness. It will be available in English and Urdu. The pre-test will assess validity and reliability, and the finalized version will be used in both pre-intervention baseline and post-intervention end-line surveys. It will have three sections: socio-demographic information, KAP related to NCDs (e.g., cardiovascular diseases, stroke, diabetes), and behavioral risk factors (e.g., diet, physical activity, smoking).

Additionally, the students’ weight and height will be measured by using calibrated weighing scales and stadiometers for accurate measurements. Weight will be measured using a digital weighing scale (Beurer PS25, Germany), and height will be measured using a portable stadiometer (Seca 213, Germany). The procedure involves requesting participating students to stand barefoot on the weighing scale, ensuring an even distribution of weight, and recording the reading to the nearest 0.1 kg. Height measurement will be taken with the student standing upright against the stadiometer, with heels, back, and head in contact with the device and readings will be recorded to the nearest 0.1 cm. Each measurement will be taken twice, and the average will be used for analysis. This approach ensures the ethical and systematic acquisition of necessary information while respecting the privacy and well-being of the participants. These measurements will be used to calculate BMI at baseline to help describe the health profile of the participating students. BMI is not an outcome of the study but will provide contextual information to support interpretation of baseline awareness and behaviors.

#### Outcomes

#### Feasibility outcomes

The feasibility of an eSHEPP will be assessed regarding recruitment, retention, and treatment fidelity [73]. The trial will be considered feasible if recruitment, retention, and treatment fidelity rates exceed 70% [74].

i. Recruitment will be measured as the percentage of participants enrolled out of the total number of invited participants at baseline phase.
ii. Retention will be assessed as the percentage of participants available for follow-up at 2 months among those recruited initially.
iii. Treatment fidelity will be defined as the proportion of the six planned health promoting sessions that are successfully conducted.

#### Potential efficacy outcome

The potential efficacy will be measured in terms of participants’ KAP at baseline and follow up of 2 months.

Knowledge of NCDs and their risk factors will be assessed with 26 questions, scored as correct or incorrect based on standard medical guidelines. The total score will be calculated, with a higher score indicating better knowledge. A score of 60% or higher will define “good knowledge,” consistent with previous KAP studies categorizing it as satisfactory [75–77].

Attitudes towards NCDs and their risk factors will be assessed with 14 Likert-scale questions. Participants will rate each item on a 3-point scale, except one rated on a 4-point scale. Scores will be summed, with a range of 16 to 49 points. A score of 60% or higher will indicate a positive attitude, consistent with previous research standards.

Practices will be assessed across three behavioral domains, such as dietary habits, physical activity, smoking, and substance abuse, using 22 items based on WHO recommendations for adolescents. For dietary habits, the participants will be classified as having dietary risk if they meet at least two of the following criteria: (i) consuming fewer than five servings of fruit per day, (ii) consuming fewer than five servings of vegetables per day, (iii) adding salt to every meal, and (iv) drinking sugar-sweetened beverages more than three days per week. Physical inactivity will be defined as not engaging in at least 60 min of moderate-intensity physical activity daily. Finally, participants who report smoking regularly or being exposed to passive smoking more than three days per week, as well as those who have consumed alcohol or used substances in the past 30 days, will be considered at risk in the substance use domain.

#### Plan of data analysis and management

Data from this pilot cRCT will be analyzed using methods that account for within-cluster correlations. Given the small number of clusters (*N* = 4), a post hoc power analysis will be conducted to assess statistical power. Descriptive statistics will summarize categorical and continuous variables, and baseline differences will be examined using chi-square tests, t-tests, or Mann-Whitney U tests as appropriate. Linear mixed-effects models will analyze continuous outcomes, while mixed-effects logistic regression will be applied to binary outcomes. Missing data will be addressed using multiple imputation if necessary. Statistical significance will be set at *p* < 0.05, and results will be presented as adjusted odds ratios (aORs) with 95% confidence intervals (CIs). Given the pilot nature of this study and the limited number of clusters, all findings will be interpreted cautiously and considered exploratory.

### Phase III: qualitative explanation - usefulness, acceptability and task technology fit of the eSHEPP

#### Study design and participants

A qualitative explanatory design will be employed. In a sequential mixed methods approach, this design provides meaningful explanations and context to the findings from the quantitative phase [78].

The study will include secondary and higher secondary school students who actively participated in the intervention and attended all six health-promoting sessions. Two FGDs will be conducted, each with eight participants: one with male students and one with female students.

#### Data collection tools and data analysis

A semi-structured interview guide will be developed using the integrated Technology Acceptance Model (TAM) and Task–Technology Fit (TTF) model, as illustrated in Fig. 4, with probes generated based on this framework. The guide will be prepared in English and translated into Urdu.

FGDs will explore students’ perspectives on the usefulness, acceptability, and task–technology fit of the eSHEPP. Discussions will be facilitated by a doctoral (PhD) student with expertise in qualitative research.

Data analysis will follow COREQ guidelines, using a hybrid approach that combines deductive analysis (predefined themes from TAM and TTF) and inductive analysis (emerging themes beyond the theoretical framework). All discussions will be audio-recorded, compiled, translated into English, and analyzed using manual thematic analysis and QSR NVivo version 10.

### Ethical considerations

Written informed consent will be obtained from all participants, or from legal guardians in the case of minors, before participation. Data will be transmitted securely among research personnel using password-protected files and a secure network. Demographic information will be aggregated and de-identified before analysis to minimize the risk of identifying individuals from their responses.

Participants’ identities will remain confidential throughout the study. Personal identifiers such as names and IP addresses will not be collected or stored.

This study has received ethical approval from The Aga Khan University Ethical Review Committee (Ref No. 2023-9277-27367) and the National Bioethics Committee of Pakistan (Ref No. 4–87/NBCR-1089/23/1896). The trial is registered at ClinicalTrials.gov (Registration No. NCT06674798, Date registered: 4 November 2024).

Any protocol changes will require approval from the project steering committee and submission to the ethics committee and trial registry before implementation. The protocol follows SPIRIT 2013 guidelines, and results will be reported in accordance with CONSORT guidelines for cRCT [79, 80].

## Discussion

This protocol presents a feasibility study of eSHEPP, a school-based digital health intervention designed to increase adolescents’ awareness and promote preventive behaviors related to NCDs. Given the increasing prevalence of NCD risk factors, such as unhealthy diets, physical inactivity, and early substance use, among Pakistani adolescents, this study examines whether a culturally tailored, technology-driven initiative can be effectively implemented within an urban school setting.

The study will assess key implementation metrics including recruitment, retention, and treatment fidelity. These feasibility indicators are essential for evaluating program viability in real-world school environments [46, 47], where barriers such as resource limitations, scheduling constraints, and the cooperation of teachers and administrators may influence success [53, 54]. In addition, student engagement with the digital application will be examined to identify which features facilitate or hinder participation [56].

Beyond implementation, the study seeks to collect early evidence on the intervention’s potential impact on students’ KAP related to NCDs and their risk factors. Outcomes will include changes in health awareness, motivation toward positive behavior change, and readiness to adopt healthier lifestyle choices following exposure to the program [81].

A key component of the study is identifying the facilitators and barriers that affect the implementation of digital health interventions in LMIC school settings. The research will explore contextual factors such as internet connectivity, staff commitment, student digital literacy, cultural norms, and technology acceptance. Understanding these elements will inform strategies for optimizing delivery and ensuring scalability [54].

Through a user-centered development process [63, 82] involving students, teachers, parents, and administrators, eSHEPP was specifically designed to align with the local educational context. This participatory approach ensures the program’s content and delivery methods are relevant, acceptable, and feasible for urban Pakistani schools. The findings from this study will generate practical guidance for designing and implementing similar school-based digital health initiatives in other resource-limited settings.

This research aims to guide education and public health policy by providing a roadmap for how digital health interventions can be adapted and scaled in low-resource settings. This study has several limitations. First, its focus on public secondary and higher secondary schools in Karachi, Pakistan restricts generalizability to private institutions, other provinces, and rural settings, where adolescents may face different digital access and health challenges. Second, the small number of clusters (*N* = 4) limits statistical power, meaning the analyses are exploratory and findings should be interpreted with caution. Despite these constraints, the study provides valuable preliminary evidence to inform future large-scale research on adolescent eHealth in LMICs.

## Conclusion

This protocol outlines the design and evaluation plan for eSHEPP, a digital, school-based intervention developed to improve adolescents’ KAP related to NCDs in urban Pakistan. The study will explore the design and content of eSHEPP, investigate barriers and facilitators to its delivery, evaluate its potential efficacy, and assess its usefulness, acceptability, and task–technology fit. Findings from this work are anticipated to guide refinements of the intervention and strengthen the evidence base for adolescent health initiatives in similar contexts.

## Supplementary Information

Below is the link to the electronic supplementary material.

Supplementary Material 1

## Data Availability

The datasets generated and/or analyzed during the current study, together with the survey instruments and qualitative interview guides, will be available from the corresponding author on reasonable request. To protect participant confidentiality, full interview transcripts will not be made publicly available; however, de-identified excerpts may be shared upon request and subject to ethical approval.

## Abbreviations

aOR: Adjusted odds ratios
cRCT: Cluster randomized controlled trial
eSHEPP: School eHealth Education Program Pakistan
FGDs: Focus Group Discussions
FIC: Fogarty International Center
ICC: Intra-cluster correlation coefficient
KIIs: Key Informant Interviews
LMICs: Low- and middle-income countries
NCDs: Non-communicable diseases
NIH: National Institute of Health
SSB: Sugar Sweetened Beverage
TAM: Technology Acceptance Model
TPB: Theory of Planned Behavior
TTF: Task Technology Fit
WHO: World Health Organization
