# Supplementary material for: School eHealth Education Program Pakistan (eSHEPP) to improve NCDs awareness in adolescents from urban Pakistan: a mixed method design protocol
Source: J Health Popul Nutr. 2025 Oct 14;44:363. doi: 10.1186/s41043-025-01097-6 (PMC12519622; doi:10.1186/s41043-025-01097-6)
Supplement: Supplementary file 1 — Supplementary Material 1 [file 41043_2025_1097_MOESM1_ESM.docx]

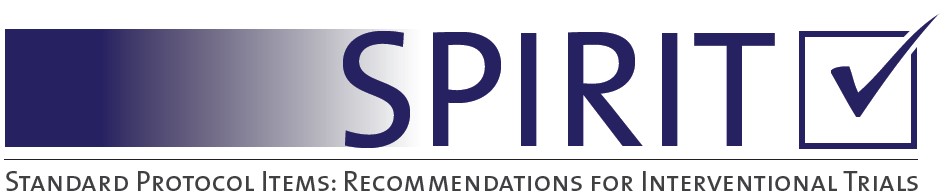


SPIRIT 2013 Checklist: Recommended items to address in a clinical trial protocol and related documents*

**Administrative information**

| Title | 1 | Descriptive title identifying the study design, population, interventions, and, if applicable, trial acronym | Page 1 |
| --- | --- | --- | --- |
| Trial registration | 2a | Trial identifier and registry name. If not yet registered, name of intended registry | Page 3 |
|  | 2b | All items from the World Health Organization Trial Registration Data Set | Primary registry and trial identifying number: ClinicaTrials.gov (ID: NCT06674798)  Date of registration in primary registry: 11/04/2024  Secondary identifying numbers: N/A  Source(s) of monetary or material support:  Primary sponsor: Fogarty International Center (FIC), National Institute of Health (NIH)  Secondary sponsor(s): N/A  Contact for public queries: [shahid.khurshid@aku.edu](mailto:shahid.khurshid@aku.edu) (Muhammad Shahid Khan)  Contact for scientific queries: [shahid.khurshid@aku.edu](mailto:shahid.khurshid@aku.edu) (Muhammad Shahid Khan)  Public title: N/A  Scientific title: School eHealth Education Program Pakistan(eSHEPP) to Improve NCDs Awareness in Adolescents from Urban Pakistan: A Mixed Method Design Protocol  Countries of recruitment: Pakistan  Health condition(s) or problem(s) studied: NCD risk behaviors- unhealthy diet, physical inactivity, smoking and alcohol use, obesity  Intervention(s): Health education (ehealth NCD awareness program) (Page 9 - 11)  Key inclusion and exclusion criteria: (Pages 8, 12, 14, 19)  Study type: Interventional (Before-after design) (Page 7-8)  Date of first enrolment: September 2024  Target sample size: 272  Recruitment status: Recruited  Primary outcome(s): Outcome (Page 17 - 18)  Key secondary outcomes: N/A |
| Protocol version | 3 | Date and version identifier | Page 1 |
| Funding | 4 | Sources and types of financial, material, and other support | Page 22 (Funding) |
| Roles and responsibilities | 5a 5b | Names, affiliations, and roles of protocol contributors  Name and contact information for the trial sponsor | Page 1 |
|  | 5c | Role of study sponsor and funders, if any, in study design; collection, management, analysis, and interpretation of data; writing of the report; and the decision to submit the report for publication, including whether they will have ultimate authority over any of these activities | The funders had no role in the design, data collection, analysis, interpretation, or writing of this protocol. |
|  | 5d | Composition, roles, and responsibilities of the coordinating centre, steering committee, endpoint adjudication committee, data management team, and other individuals or groups overseeing the trial, if applicable (see Item 21a for data monitoring committee) | The coordinating centre, based at the Department of Medicine, Aga Khan University, will manage overall trial coordination, including site oversight, staff training, communication, and regulatory compliance.  The steering committee—comprising the PI, three co-investigators, and one external public health expert—will provide scientific oversight, ensure protocol adherence, and review major trial events. It will meet quarterly.  An independent endpoint adjudication committee (one clinical expert, one statistician), blinded to group allocation, will validate primary outcome events to ensure objectivity.  The data management team at the Clinical Trials Unit, Aga Khan University, will oversee secure data entry, validation, cleaning, and confidentiality compliance.  Given the low-risk nature of the intervention and the limited scale of the trial, a Data Monitoring Committee (DMC) has not been established. Safety and trial conduct will be monitored by the principal investigator and the steering committee. |
| **Introduction** |  |  |  |
| Background and rationale | 6a | Description of research question and justification for undertaking the trial, including summary of relevant studies (published and unpublished) examining benefits and harms for each intervention | Page 4-7 (Introduction) |
|  | 6b | Explanation for choice of comparators | Page 4-7 (Introduction) |
| Objectives | 7 | Specific objectives or hypotheses | Page 7 (Aims) |
| Trial design | 8 | Description of trial design including type of trial (eg, parallel group, crossover, factorial, single group), allocation ratio, and framework (eg, superiority, equivalence, noninferiority, exploratory) | Page 7 & 8 (Study Design) |

**Methods: Participants, interventions, and outcomes**

| Study setting | 9 | Description of study settings (eg, community clinic, academic hospital) and list of countries where data will be collected. Reference to where list of study sites can be obtained | 8 (Study setting and study participants) |
| --- | --- | --- | --- |
| Eligibility criteria | 10 | Inclusion and exclusion criteria for participants. If applicable, eligibility criteria for study centres and individuals who will perform the interventions (eg, surgeons, psychotherapists) | Overall study: Page 2 & 8 Qualitative : Page 12 Quantitative: Page 13 -14 Qualitative: Page 19 |
| Interventions | 11a | Interventions for each group with sufficient detail to allow replication, including how and when they will be administered | Page 9-11 & 14 |
|  | 11b | Criteria for discontinuing or modifying allocated interventions for a given trial participant (eg, drug dose change in response to harms, participant request, or improving/worsening disease) | Intervention is educational with no known risks requiring discontinuation or concomitant care limitations. |
|  | 11c | Strategies to improve adherence to intervention protocols, and any procedures for monitoring adherence (eg, drug tablet return, laboratory tests) | Page 14 (eSHEPP Intervention) |
|  | 11d | Relevant concomitant care and interventions that are permitted or prohibited during the trial | N/A |
| Outcomes | 12 | Primary, secondary, and other outcomes, including the specific measurement variable (eg, systolic blood pressure), analysis metric (eg, change from baseline, final value, time to event), method of aggregation (eg, median, proportion), and time point for each outcome. Explanation of the clinical relevance of chosen efficacy and harm outcomes is strongly recommended | Page 3 & 17- 18 (Outcomes) |
| Participant timeline | 13 | Time schedule of enrolment, interventions (including any run-ins and washouts), assessments, and visits for participants. A schematic diagram is highly recommended (see Figure) | Additional file 1 |
| Sample size | 14 | Estimated number of participants needed to achieve study objectives  and how it was determined, including clinical and statistical assumptions supporting any sample size calculations | Page 14-15 (Sample size) |
| Recruitment | 15 | Strategies for achieving adequate participant enrolment to reach target sample size | Page 16 (Data collection tool and procedure) |

**Methods: Assignment of interventions (for controlled trials)**

| Allocation: |  |  |  |
| --- | --- | --- | --- |
| Sequence generation | 16a | Method of generating the allocation sequence (eg, computer generated random numbers), and list of any factors for stratification. To reduce predictability of a random sequence, details of any planned restriction (eg, blocking) should be provided in a separate document that is unavailable to those who enroll participants or assign interventions | Page 14 (Randomization and blinding) & Fig 5 |
| Allocation  concealment mechanism | 16b | Mechanism of implementing the allocation sequence (eg, central telephone; sequentially numbered, opaque, sealed envelopes), describing any steps to conceal the sequence until interventions are assigned | Page 14 (Randomization and blinding), 14 (eSHEPP Intervention) & Fig 5 |
| Implementation | 16c | Who will generate the allocation sequence, who will enrol participants, and who will assign participants to interventions | Page 14 (Randomization and blinding) |
| Blinding  (masking) | 17a | Who will be blinded after assignment to interventions (eg, trial participants, care providers, outcome assessors, data analysts), and how | Page 14 (Randomization and blinding) |
|  | 17b | If blinded, circumstances under which unblinding is permissible, and procedure for revealing a participant’s allocated intervention during the trial | N/A |

**Methods: Data collection, management, and analysis**

| Data collection methods | 18a | Plans for assessment and collection of outcome, baseline, and other trial data, including any related processes to promote data quality (eg, duplicate measurements, training of assessors) and a description of study instruments (eg, questionnaires, laboratory tests) along with their reliability and validity, if known. Reference to where data collection forms can be found, if not in the protocol | Qualitative : Page 12-13 Quantitative: Page 16 Qualitative: Page 19 |
| --- | --- | --- | --- |
|  | 18b | Plans to promote participant retention and complete follow-up, including list of any outcome data to be collected for participants who discontinue or deviate from intervention protocols | Retention strategies will include reminder calls and flexible session timing. We will track and report any deviations or dropouts –  Page 17 & 18 (Feasibility outcomes) |
| Data management | 19 | Plans for data entry, coding, security, and storage, including any related processes to promote data quality (eg, double data entry; range checks for data values). Reference to where details of data management procedures can be found, if not in the protocol | Page 18 (Plan of data analysis and management) |
| Statistical methods | 20a | Statistical methods for analysing primary and secondary outcomes. Reference to where other details of the statistical analysis plan can be found, if not in the protocol | Qualitative: Page 12-13 Quantitative: Page 18 Qualitative: Page 19 |
|  | 20b | Methods for any additional analyses (eg, subgroup and adjusted analyses) | N/A |
|  | 20c | Definition of analysis population relating to protocol non-adherence (eg, as randomised analysis), and any statistical methods to handle missing data (eg, multiple imputation) | Page 18 (Plan of data analysis & management) |

**Methods: Monitoring**

| Data monitoring | 21a | Composition of data monitoring committee (DMC); summary of its role and reporting structure; statement of whether it is independent from the sponsor and competing interests; and reference to where further details about its charter can be found, if not in the protocol. Alternatively, an explanation of why a DMC is not needed | N/A |
| --- | --- | --- | --- |
|  | 21b | Description of any interim analyses and stopping guidelines, including who will have access to these interim results and make the final decision to terminate the trial | N/A |
| Harms | 22 | Plans for collecting, assessing, reporting, and managing solicited and spontaneously reported adverse events and other unintended effects of trial interventions or trial conduct | Given that this is a low-risk educational intervention, consider adding: “No adverse events are expected; any issues will be recorded and reviewed by the PI and steering committee.” |
| Auditing | 23 | Frequency and procedures for auditing trial conduct, if any, and whether the process will be independent from investigators and the sponsor | Quantitative: Page 10 -11 (Proposed content of School eHealth Education Program Pakistan (eSHEPP)) & 14 (eSHEPP Intervention)  Qualitative: Page 19 (Data collection tools and data analysis) |

**Ethics and dissemination**

| Research ethics approval | 24 | Plans for seeking research ethics committee/institutional review board (REC/IRB) approval | Page 22 (Ethics approval) |
| --- | --- | --- | --- |
| Protocol amendments | 25 | Plans for communicating important protocol modifications (eg, changes to eligibility criteria, outcomes, analyses) to relevant parties (eg, investigators, REC/IRBs, trial participants, trial registries, journals, regulators) | Any protocol modifications will be submitted to the IRB and communicated to study staff and trial registries as required. |
| Consent or assent | 26a | Who will obtain informed consent or assent from potential trial participants or authorised surrogates, and how (see Item 32) | Informed consent will be obtained by trained research staff prior to participation. Written consent forms will be used. |
|  | 26b | Additional consent provisions for collection and use of participant data and biological specimens in ancillary studies, if applicable | N/A |
| Confidentiality | 27 | How personal information about potential and enrolled participants will be collected, shared, and maintained in order to protect confidentiality before, during, and after the trial | Participant information will be kept strictly confidential. Data will be collected using unique ID codes, with personal identifiers stored separately in a secure, password-protected system accessible only to authorized staff. Hard copies will be kept in locked cabinets, and electronic data on encrypted servers.  Only de-identified data will be used for analysis and reporting. All personal identifiers will be excluded from publications. De-identified data will be securely archived for five years, while identifiable data will be destroyed after the retention period unless extended use is approved. |
| Declaration of interests | 28 | Financial and other competing interests for principal investigators for the overall trial and each study site | Page 22 (Competing interests) |
| Access to data | 29 | Statement of who will have access to the final trial dataset, and disclosure of contractual agreements that limit such access for investigators | The final trial dataset will be accessible only to the principal investigator and authorized research team members. There are no contractual limitations on access. |
| Ancillary and post-trial care | 30 | Provisions, if any, for ancillary and post-trial care, and for compensation to those who suffer harm from trial participation | N/A |
| Dissemination policy | 31a | Plans for investigators and sponsor to communicate trial results to participants, healthcare professionals, the public, and other relevant groups (eg, via publication, reporting in results databases, or other data sharing arrangements), including any publication restrictions | Trial results will be published in peer-reviewed journals and presented to schools and public health stakeholders. Page 3 (Ethics and dissemination) Page 20 (Ethical considerations). |
|  | 31b | Authorship eligibility guidelines and any intended use of professional writers | No professional writers are used. |
|  | 31c | Plans, if any, for granting public access to the full protocol, participant level dataset, and statistical code | Page 22 (Availability of data and materials) |

**Appendices**

| Informed consent materials | 32 | Model consent form and other related documentation given to participants and authorised surrogates | Will be provided on request |
| --- | --- | --- | --- |
| Biological specimens | 33 | Plans for collection, laboratory evaluation, and storage of biological specimens for genetic or molecular analysis in the current trial and for future use in ancillary studies, if applicable | N/A |

*It is strongly recommended that this checklist be read in conjunction with the SPIRIT 2013 Explanation & Elaboration for important clarification on the items. Amendments to the protocol should be tracked and dated. The SPIRIT checklist is copyrighted by the SPIRIT Group under the Creative Commons “[Attribution-NonCommercial-NoDerivs 3.0 Unported”](http://www.creativecommons.org/licenses/by-nc-nd/3.0/) license.
